# An Image-Based Drug Susceptibility Assay Targeting the Placental Sequestration of *Plasmodium falciparum*-Infected Erythrocytes

**DOI:** 10.1371/journal.pone.0041765

**Published:** 2012-08-29

**Authors:** Min-Je Ku, Fernando de M. Dossin, Michael A. E. Hansen, Auguste Genovesio, Lawrence Ayong, Lucio H. Freitas-Junior

**Affiliations:** 1 Center for Neglected Diseases Drug Discovery (CND3), Institut Pasteur Korea, Seongnam-si, Gyeonggi-do, South Korea; 2 Center for Core Technologies-Image Mining, Institut Pasteur Korea, Seongnam-si, Gyeonggi-do, South Korea; State University of Campinas, Brazil

## Abstract

Placental malaria is a significant cause of all malaria-related deaths globally for which no drugs have been developed to specifically disrupt its pathogenesis. To facilitate the discovery of antimalarial drugs targeting the cytoadherence process of *Plasmodium*-infected erythrocytes in the placenta microvasculature, we have developed an automated image-based assay for high-throughput screening for potent cytoadherence inhibitors *in vitro*. Parasitized erythrocytes were drug-treated for 24 h and then allowed to adhere on a monolayer of placental BeWo cells prior to red blood cell staining with glycophorin A antibodies. Upon image-acquisition, drug effects were quantified as the proportion of treated parasitized erythrocytes to BeWo cells compared to the binding of untreated iRBCs. We confirmed the reliability of this new assay by comparing the binding ratios of CSA- and CD36-panned parasites on the placental BeWo cells, and by quantifying the effects of chondroitin sulfate A, brefeldin A, and artemisinin on the binding. By simultaneously examining the drug effects on parasite viability, we could discriminate between cytoadherence-specific inhibitors and other schizonticidal compounds. Taken together, our data establish that the developed assay is highly suitable for drug studies targeting placental malaria, and will facilitate the discovery and rapid development of new therapies against malaria.

## Introduction

*Plasmodium falciparum* is responsible for the most severe forms of human malaria that include cerebral malaria, pregnancy-associated (placental) malaria, and acute anemia.

A major aspect of the virulence of *P. falciparum* derives from the ability of parasitized erythrocytes to adhere to different endothelial cell types in the deep vasculature of the body, thus resulting in a sequestration of the parasites away from splenic clearance [1], [2], [3]. The specificity of malaria parasite cytoadherence is mediated by variants of the parasite-specific adhesin, ***P*** *.* ***f*** *alciparum* **e**rythrocyte **m**embrane **p**rotein 1 (PfEMP1) that are exported and assembled on the infected host cell surface where they interact with diverse cellular receptors in the microvasculature [4], [5]. Whereas parasite sequestration in the peripheral microvasculature is associated with parasitized erythrocytes that bind to CD36, ICAM-1, VCAM or E-selectin receptors, sequestration in the placenta mainly involves chondroitin sulfate A (CSA) that is abundantly expressed by placental syncytiotrophoblasts [1], [6], [7]. Furthermore, evidence from targeted gene disruption studies have established that the PfEMP1 variant var2CSA is the main ligand mediating the cytoadherence process against placental CSA receptors [8], [9], [10], [11]. In support of these observations, var2CSA-dependent binding of parasitized erythrocytes to placental BeWo cells can be efficiently inhibited by pretreatment with soluble CSA proteins [11]. This suggests that a disruption of var2CSA protein export and assembly on the erythrocyte surface, or inhibition through chemotherapy of its interaction with placental CSA receptors could limit the disease severity reversing the pathophysiology of placental malaria. However, despite the availability of several drug compounds targeting the asexual development and growth of malaria parasites *in vitro* and *in vivo*, none of the current antimalarial drugs and those in clinical development is able to protect against placental malaria. For example, post-mortem studies of severe malaria in previously treated patients often show high levels of infected erythrocytes bound to the microvasculature in spite of clearance of the peripheral parasitaemia [12], [13]. These observations strongly underscore the urgent need for new antimalarial drugs targeting the cytoadherence process of malaria parasites, particularly in high-risk pregnancy cases.

Here, we describe the development of a novel image-based assay for the high-throughput screening and identification of small molecule inhibitors of parasitized erythrocyte cytoadherence in the placenta during pregnancy.

## Materials and Methods

### *P. falciparum* and BeWo cell cultures

*Plasmodium falciparum* FCR3 strain was maintained in RPMI 1640 media supplemented with *L*-glutamine, 25 mM HEPES, sodium bicarbonate, 0.5% Albumax, 0.1 mM hypoxanthine and 16 µM thymidine in human O^+^ erythrocytes [14]. To enrich for the PfEMP1^var2CSA^ expressing phenotypes, parasitized erythrocytes were panned once every three weeks, and also before use in our binding assays against a monolayer of BeWo cells in culture dishes [8]. Additionally, parasites were synchronized by two sequential treatments with 5% sorbitol at 10 hours interval, and cultivated for at least one complete cycle prior to the drug assays with early ring stage parasites (∼6 hpi).

The human choriocarcinoma placental cell line (BeWo) was obtained from the American Type Culture Collection (ATCC CCL-98) and grown in Ham's F12 medium supplemented with *L*-glutamine (Invitrogen) and 10% fetal bovine serum (Gibco). For the cytoadherence assays, adherent BeWo cells were detached using trypsin-EDTA (Gibco) and washed with assay-complete medium (ACM) comprising Ham's F12 medium supplemented with 4.7% human serum (Sigma), 0.23% Albumax I (Gibco) and 0.7% fetal bovine serum. The cells were then seeded at a density of 2000 BeWo cells per 50 µl culture in 384-well plates (Greiner), and grown for 4 days at 37°C to achieve approximately 80% confluency prior to the cytoadherence assays.

### Cytoadherence assay and image acquisition

Panned *P. falciparum* FCR3-infected erythrocytes were diluted in complete culture media (50 µl per 384-well) to a final parasitaemia of 6% and hematocrit 2%, and then cultivated with or without drugs for 24 h at 37°C. After mixing by vortexing at 1700 rpm for 45 seconds (Mixmate), 5 µl of the samples were transferred onto a monolayer of BeWo cells in a second microtiter plate and incubated for 1 h at 37°C to allow for binding of the infected erythrocytes. Unbound erythrocytes were washed three times with assay complete media using an EL406 combination washer (Biotek), and the attached cells fixed with 4% paraformaldehyde at RT for 15 minutes. This was followed by nucleic acid staining with Syto60 (Molecular Probes) diluted in PBS (1∶4000) and erythrocyte membrane labeling with anti-glycophorin A FITC-conjugated antibody (Caltag Laboratories) at a 1∶1000 dilution in PBS. The plates were washed again and imaged using an ImageXpress Ultra automated-confocal microscope (Molecular Devices). Four images (2000 pixel×2000 pixel each) were acquired from each test well using a 20×-magnifying lens, and analyzed using customized algorithms that were developed in-house.

### Image mining algorithms and data analysis

To quantitatively determine the effect of small molecule inhibitors of *P. falciparum* cytoadherence to BeWo cells, we developed specific algorithms capable of measuring the proportion of overlapping BeWo cell area with bound parasitized erythrocytes. We assumed that all parasitized erythrocytes are of the same sizes and that the proportion of BeWo cell area occupied by the bound erythrocytes directly correlates with the number of adhering erythrocytes. We confirmed such correlations by measuring the proportion of overlapping infected red blood cell area per BeWo cell area with increasing amounts (parasitaemia) of panned erythrocytes. For both the attached RBCs (green fluorescence channel) and BeWo cells (red fluorescence channel), a Gaussian low-pass filter [15], [16] was used for noise filtering whereas adaptive thresholding was used for the cell segmentations. This adaptive threshold was based on a k-means clustering algorithm that separates image pixels into either foreground (BeWo or iRBC) or background [16], [17]. The above-described algorithm was then implemented as a plugin (programming language C-Sharp) to Institut Pasteur Korea's High Content Screening platform that is currently accessible only to authorized users (cf Moon and Genovesio, 2008) [18].

### Drug effects on parasite cytoadherence and viability

To validate the assay protocol, we investigated the effects of the cytoadherence competitive inhibitor chondroitin sulfate A (CSA), the protein transport inhibitor brefeldin A (BFA), and the antimalarial compound artemisinin (ART) on cytoadherence to the BeWo cells and parasite growth *in vitro*. Dose-response experiments were designed with final concentration ranges from 1 mg/ml to 50 µg/ml for CSA (3-fold dilution), 160 µM to 313 nM for BFA (2-fold dilution), and 400–0.78 nM for ART (2-fold dilution) in a 384-well plate. Panned parasites at early ring stages were then added and grown for 24 h to allow for the expression and assembly of cytoadherence factors on the infected RBCs. Five microliters (5 µl) of each culture was then transferred onto a previously prepared plate with BeWo cells and analyzed for cytoadherence as described above. To assess the effects of each compound on the parasite growth, plates with the remaining culture (45 µl per well) were incubated for a further 24 h to allow for schizont egress and invasion. The parasite growth relative to non-treated controls (wells containing DMSO at 0.5%) was then determined using the pLDH assay [16], and their EC_50_ values determined using GraphPad Prism 5.0. All assays were done in triplicates and the calculated standard deviations used to assess the assay reproducibility. Additionally, z-factors which inform on the reliability of each test procedure [19] were determined for both the cytoadherence and viability assays using DMSO as positive control and CSA (1 mg/ml) or ART (400 nM), respectively, as negative controls. To confirm the involvement of CSA receptor binding in the assays, CSA and CD36-panned erythrocytes [8], [20] were analyzed at equal parasitaemias (6% parasitaemia) for binding to the attached BeWo cells.

## Results

**Figure 1:**
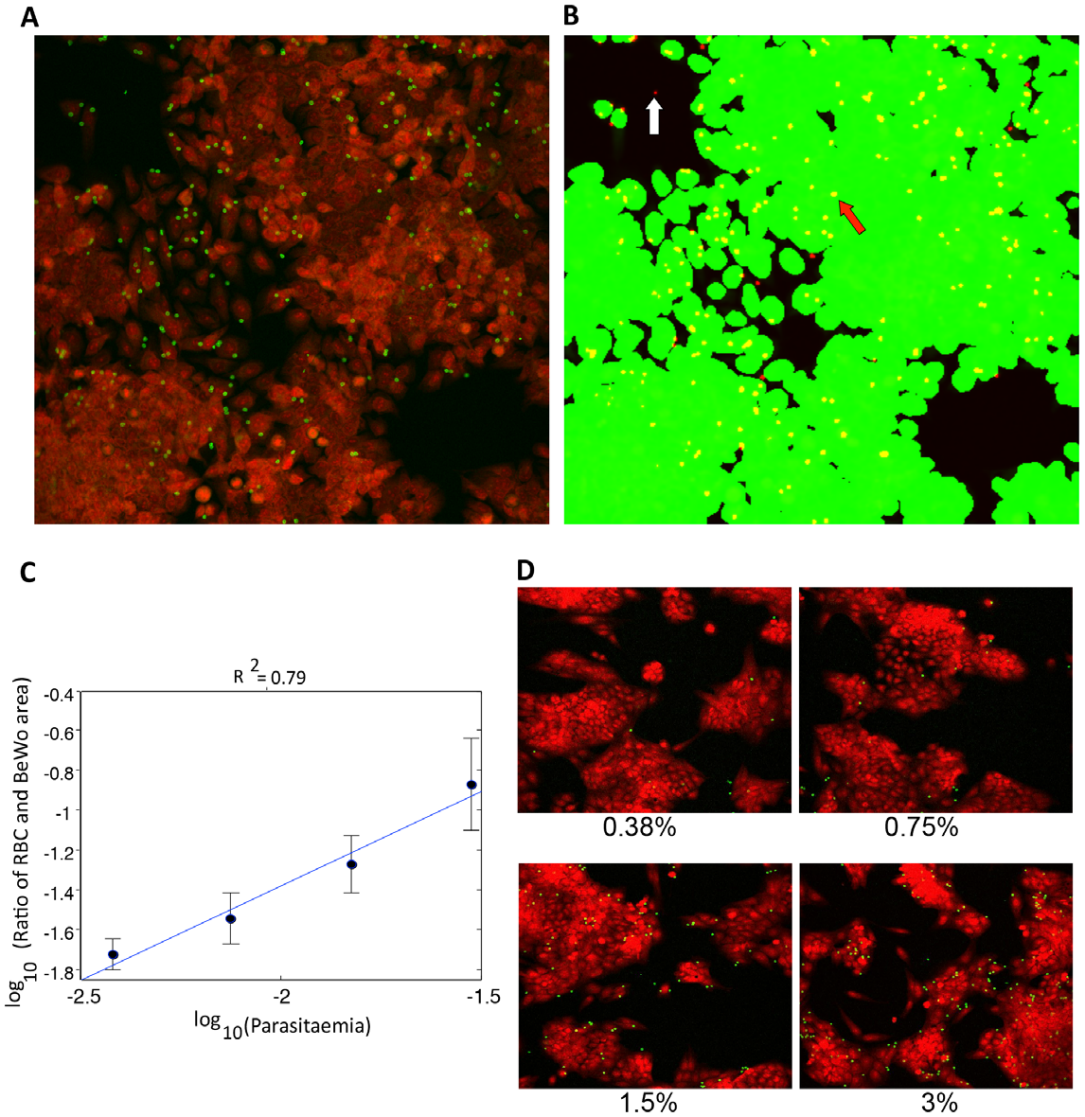
Automated detection and quantification of parasitized erythrocytes binding to plated BeWo cells. (**A**) Representative fluorescence image field showing BeWo cells in red (Syto 60-stained) and bound erythrocytes in green (anti-glycorphorin A-FITC conjugates labeled). Areas in black represent regions with no attached BeWo cells. (**B**) Image segmentation of stained BeWo cell areas (green) and erythrocyte-bound regions (yellow, the red arrow). Non-overlapping erythrocytes are indicated in red (the white arrow). Following the segmentation, binding ratios are calculated as the mean proportion of yellow areas to the green areas per microtiter well (four image fields×2000×2000 pixels). (**C**) Plot of binding ratios as a function of increasing parasitaemia, showing a strong correlation (R^2^ = 0.79 [21]) between the two parameters. (**D**) Representative image fields showing an increased proportion of bound erythrocytes with increasing parasitaemia (compare the number of green spots in the image fields denoted 0.38% and 3%). The numbers below each image field represents the culture parasitaemia used in the binding.

**Figure 2:**
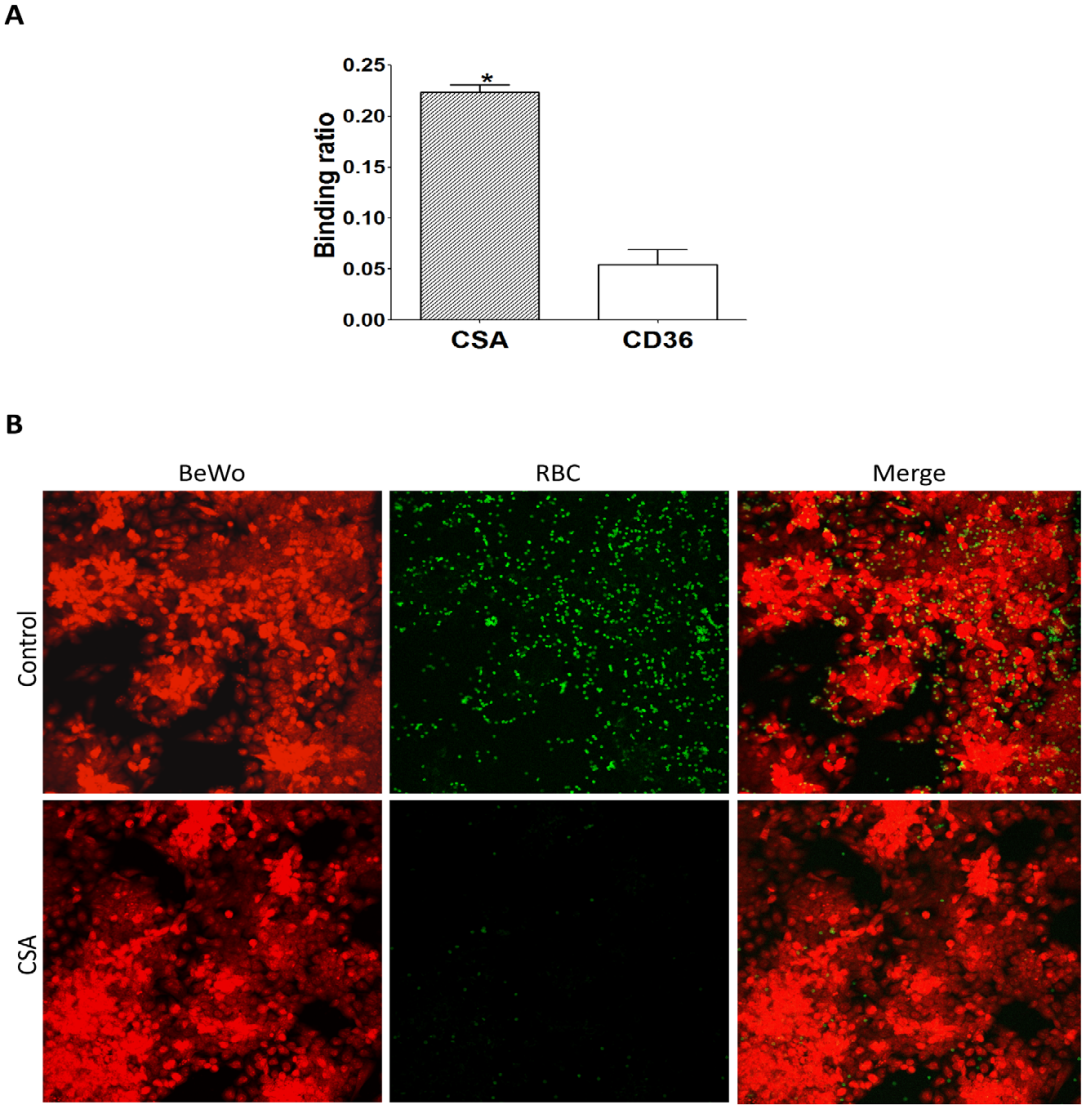
Specific involvement of CSA receptors in binding of the parasitized erythrocytes to plated BeWo cells. (**A**) Significantly increased binding (Student t-test, *P = 0.004*) of CSA-panned erythrocytes (bar labeled “CSA”) to BeWo cells when analyzed in parallel and at equal parasitaemia with CD36-panned cells (bar labeled “CD36”). (**B**) Representative image fields showing a near-complete inhibition of binding between BeWo-panned erythrocytes and the plated BeWo cells in the presence of soluble CSA (1 mg/ml). Control wells were treated with DMSO at a 0.5% final concentration.

To facilitate the discovery of new antimalarial drugs targeting the cytoadherence process of *Plasmodium*-infected erythrocytes during pregnancy, we have developed an image-based drug susceptibility assay suitable for high-throughput screening of diverse chemical libraries. This assay was designed for identifying drug compounds capable of inhibiting either the export (and assembly) of malaria parasite cytoadherence molecules on the infected erythrocyte surface, or their interactions with cellular receptors in the placenta microvasculature. Parasitized erythrocytes at the late developmental stages were layered on a monolayer of placental BeWo cells and allowed to bind for 1 h at 37°C. The attached erythrocytes were then labeled with glycophorin A antibody conjugates whereas the underlying BeWo cells were stained with the nucleic acid dye Syto60 for fluorescence microscopy imaging. For an unbiased quantification of binding ratios, specific image mining algorithms were developed (Fig. 1A–B) and validated for its performance in detecting the effects of two known cytoadherence inhibitors, CSA and BFA, and the antimalarial drug artemisinin. As shown in Fig. 1C–D, a positive correlation [21] of 0.79 was obtained between the binding ratios (proportion of placental BeWo cells with attached erythrocytes) as determined by the developed algorithms and the culture parasitaemias, thus indicating a high performance of the assay in detecting changes in erythrocyte binding capabilities. To confirm the involvement of host CSA receptors in binding of the infected erythrocyte to placental BeWo cells, CSA and CD36-panned erythrocytes were allowed to bind on the BeWo cells and their binding ratios were compared. As shown in Fig. 2A, the CSA-panned erythrocytes exhibited over 4-fold increase in binding to the placental cells when compared to the CD36-panned cells (p-value of 0.004 [22]). Likewise, binding of the BeWo-panned erythrocytes to the BeWo containing plates was highly sensitive to the presence of soluble CSA in the binding assay (Fig. 2B), thus indicating a high specificity of the binding interactions in our developed system. Together, these findings strongly indicate that the assay is highly suitable and relevant for drug studies specifically targeting the cytoadherence of *P. falciparum*-infected erythrocytes in the placenta.

**Figure 3:**
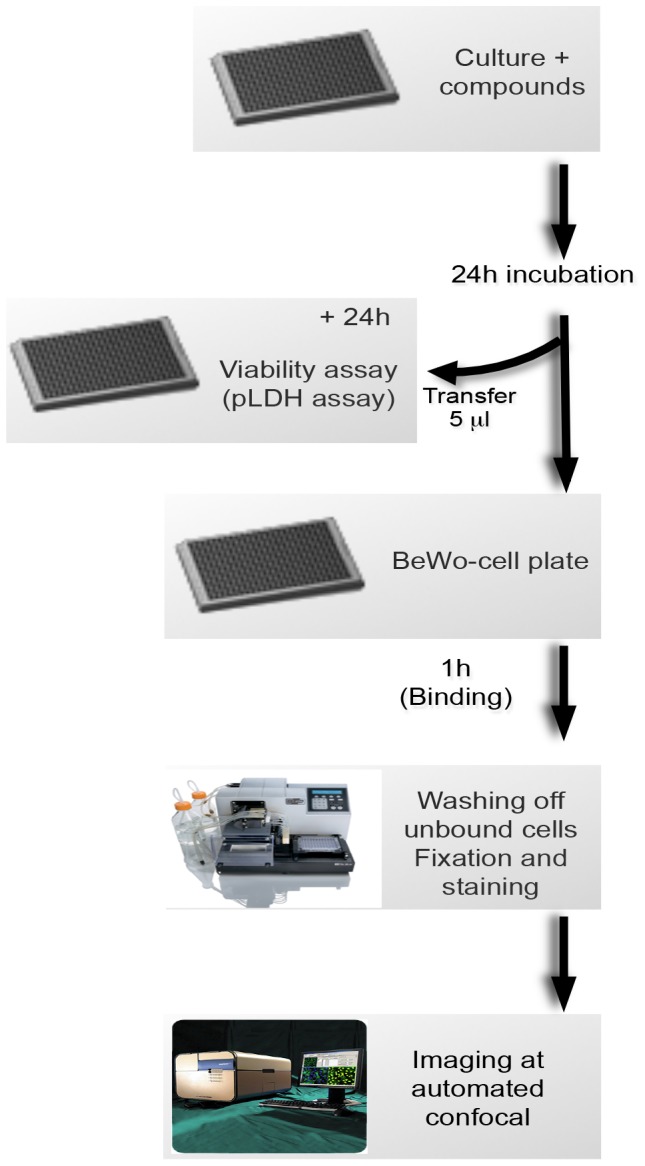
Image-based Plasmodium cytoadherence assay design. Panned *P. falciparum* FCR3 parasites (∼6-hpi) are drug-treated for 24 hours in a 384-well plate. Next, the cultures are mixed and 5 µl transferred into corresponding wells of a second plate with BeWo cells at >80% confluency. Meanwhile, the remainder 45 µl culture is further cultivated for 24 hours to complete one cell division cycle and then analyzed by the pLDH viability assay. Following a one hour binding reaction, unbound cells are washed, followed by a 15 min fixation with 4% paraformaldehyde and staining with anti-glycophorin A antibodies (bound erythrocytes) and Syto 60 (BeWo cells and parasitized erythrocytes). Stained cells are then imaged and analyzed using customized image-mining algorithms that were developed in this study.

**Figure 4:**
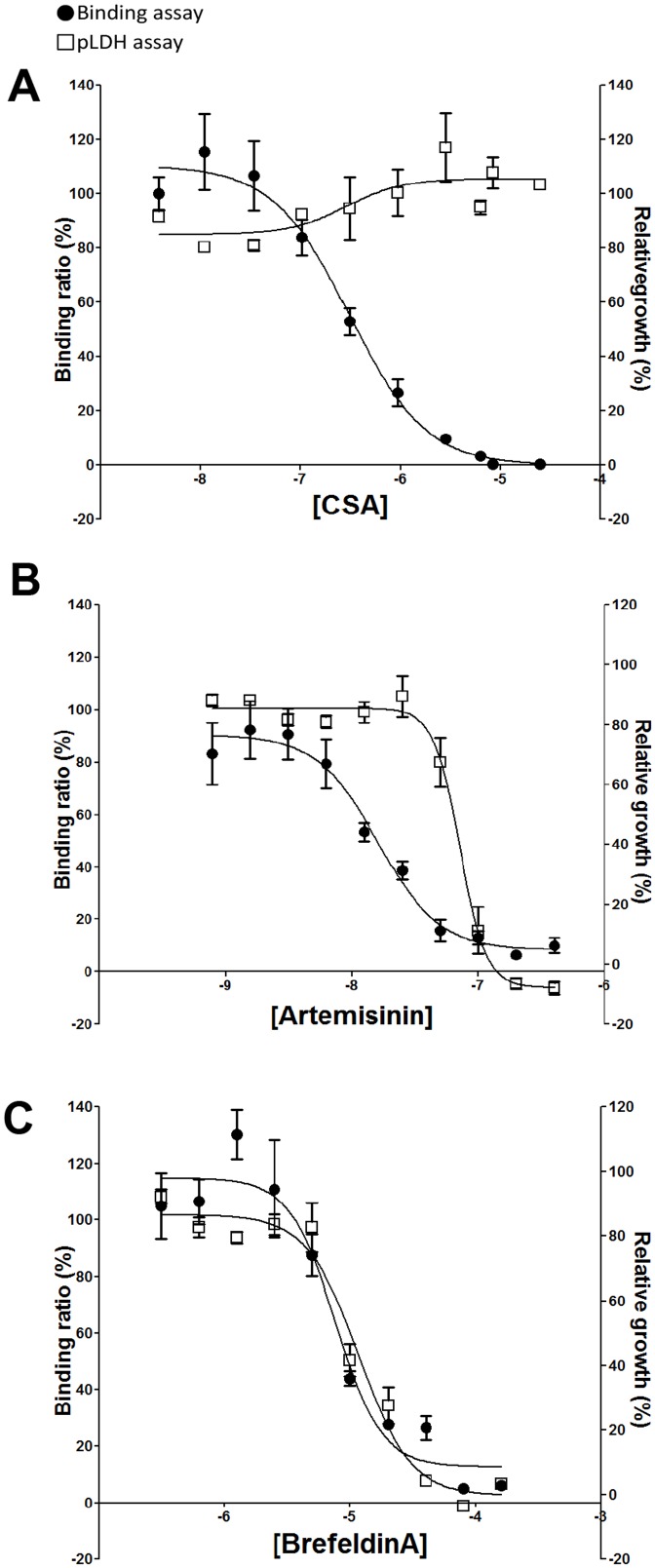
Determination of drug effects on BeWo cell binding of parasitized erythrocytes. Dose-response experiments were done in parallel using the developed assay (solid points) and pLDH growth assay (open rectangles) to assess the effects of CSA (**A**), artemisinin (**B**), and brefeldin A (**C**) on the binding to BeWo cells (image-based assay) and viability (pLDH assay). Data show a specific inhibition of cytoadherence but not parasite growth by CSA unlike artemisinin and BFA, which inhibited both binding and parasite growth with increasing drug concentrations.

To validate the assay for the quantitative assessment of diverse drug effects *in vitro*, dose-response experiments were done using the cytoadherence inhibitor CSA, as well as the protein transport inhibitor BFA, and the schizonticide artemisinin. In parallel, parasite viability assays were done with all three agents as described in the “Materials and Methods” section and Fig. 3 in order to distinguish between potentially cytotoxic agents from the cytoadherence-specific inhibitors. As shown in Fig. 4A–C, binding of the BeWo-panned infected erythrocytes to the plated BeWo cells was highly sensitive to increasing concentrations of all three agents with half maximum effective concentration (EC_50_) values of 12 µg/ml for CSA, 8 µM for BFA and 16 nM for artemisinin. A comparison of parasite viability in the presence of the different drug concentrations following an extended period of additional 24 hours indicates that only artemisinin and BFA were toxic to the parasite growth with EC_50_ values of 72 nM and 12 µM, respectively. These data indicate that the observed artemisinin or BFA inhibition of parasitized erythrocyte binding to the BeWo cells presumably occurred due to the drug effects on parasite growth, and that soluble CSA is a specific inhibitor of *Plasmodium* cytoadherence. Taken together, the data suggest that our combined assay is capable of distinguishing between cytoadherence-specific agents and cytotoxic compounds that might influence the hit-selection process. To further validate our developed assay system in terms of its reproducibility and/or hit detection accuracy, replicate experiments (n = 192 wells/plate×3 plates) were done using untreated parasites as positive controls or CSA (1 mg/ml)-treated cultures as negative controls. Using the calculated mean binding ratios and standard deviations from both controls, a z′ value of 0.4 was then determined for the new assay (data not shown). These data suggest that the developed assay is reliable for the use in high-throughput screening of diverse compound libraries.

## Discussion

To facilitate the rapid discovery and development of new antimalarials targeting the cytoadherence of *Plasmodium*-infected erythrocytes in the placenta microvasculature, we have developed a robust and technically simple image-based phenotypic assay for malaria based on the use of placental BeWo cells in a 384-well plate format. Specific image-mining algorithms were developed for automated quantification of binding ratios, and for accurate assessment of various drug effects. As evidenced by i) the high correlation coefficient (R^2^) of 0.79 between the determined binding ratios and parasitized erythrocyte ratios (parasitaemias), ii) the high sensitivity of our assay (detection limit <0.38% parasitaemia), and the calculated z′ value of 0.4, our developed assay is suitable for high-throughput drug studies targeting placental malaria. Additionally, our assay represents a more relevant drug susceptibility system for placental malaria when compared to assays with immobilized CSA since the herein used assay utilizes whole cells that were derived from human placental tissues [6]. BeWo cells are heterogenous cells expressing several diverse parasite cytoadhesion receptors including CSA in abundant amounts, and to a lesser extent, intercellular adhesion molecule-1 (ICAM-1) and neonatal Fc receptors, all of which are capable of interacting with *P. falciparum*-parasitized erythrocytes [8], [23], [24]. Unlike peripheral cell-types such as in the brain microvasculature, placental BeWo cells do not express other known cytoadhesion receptors including CD36, CD31, E-selectin and vascular cell adhesion molecule-1 (VCAM-1) [8], [11]. In support of the absence of CD36 receptors on BeWo cells, parasitized erythrocytes that were panned against immobilized CD36 proteins did not significantly bind to the adherent BeWo cells when compared to the CSA-panned cells (Fig. 2A). These observations suggest that the developed assay is highly specific and suitable for drug studies targeting CSA-dependent binding in the placenta.

To further validate the developed assay for use in anti-cytoadhesion drug studies, we investigated its performance in quantifying the inhibitory effects of soluble CSA, the protein transport inhibitor BFA, and the fast-acting antimalarial drug artemisinin [25], [26]. Consistent with a predominant involvement of the CSA receptor in binding to the BeWo cells [8], [11], [27], soluble CSA efficiently inhibited the cytoadherence process with an EC_50_ value of ∼12 µg/ml (Fig. 2B and 4A). This EC_50_ value is consistent with the activity range of CSA using FCR3-parasitized erythrocytes and BeWo cells [11], [27]. However, the obtained EC_50_ value is significantly high when compared to published activity data using immobilized CSA (∼0.2 µg/ml) [28], or using other parasite strains (approximately 70% inhibition of CS2 binding with 10 µg/ml CSA) [27]. These observations suggest that BeWo cells presumably bind the parasitized erythrocytes with much higher avidity than would be obtained with purified receptors, and that the associated binding interactions are strain-dependent. In contrast to the effects of artemisinin and BFA that inhibited the parasite growth, CSA was non-cytotoxic suggesting that CSA is a specific inhibitor of placental cytoadherence processes. Meanwhile, the observed binding inhibition by artemisinin and BFA in this study presumably resulted from the drug effects on the parasite growth as previously reported [12], [29]. Taken together, our findings strongly indicate that the developed assay, when combined with a growth inhibition assay, can reliably discriminate between cytoadherence-specific effects and parasite cytotoxicity effects. Interestingly, both BFA (cytoadherence protein export inhibitor) and CSA (cytoadherence inhibitor) inhibited the cytoadherence process in our assay [30]. This suggests that compounds with anti-adherent activities may function either in the secretory pathway of cytoadherence molecules, or by direct interference with the cytoadherence process as observed with CSA. This new assay represents a significant advancement in antimalarial drug discovery that will facilitate the development of novel anti-adhesive therapies against the disease. A unique attribute of the assay is in the customized algorithms used in quantifying the parasite binding ratios. These algorithms are easy to develop and can be readily replicated in any screening facility that is equipped with a medium-size computer laboratory and a plate microscope for imaging.
